# The Roles of Matricellular Proteins in Oncogenic Virus-Induced Cancers and Their Potential Utilities as Therapeutic Targets

**DOI:** 10.3390/ijms18102198

**Published:** 2017-10-21

**Authors:** Naoyoshi Maeda, Katsumi Maenaka

**Affiliations:** 1 Center for Research and Education on Drug Discovery, Faculty of Pharmaceutical Sciences, Hokkaido University, Kita-12, Nishi-6, Kita-ku, Sapporo 060-0812, Japan; 2 Laboratory of Biomolecular Science, Faculty of Pharmaceutical Sciences, Hokkaido University, Kita-12, Nishi-6, Kita-ku, Sapporo 060-0812, Japan; maenaka@pharm.hokudai.ac.jp

**Keywords:** matricellular proteins, oncogenic viruses, osteopontin (OPN), periostin (POSTN), secreted protein acidic and rich in cysteine (SPARC), tenascin-C (TNC), thrombospondin (TSP), tumor microenvironment

## Abstract

Matricellular proteins differ from other classical extracellular matrix proteins; for instance, they are transiently expressed as soluble proteins rather than being constitutively expressed in pathological conditions, such as acute viral infections. Accumulating studies have revealed that matricellular proteins, including osteopontin and tenascin-C, both of which interact with integrin heterodimers, are involved in inflammatory diseases, autoimmune disorders, and cancers. The concentrations of these matricellular proteins are elevated in the plasma of patients with certain types of cancers, indicating that they play important roles in oncogenesis. Chronic viral infections are associated with certain cancers, which are distinct from non-viral cancers. Viral oncogenes play critical roles in the development and progression of such cancers. It is vital to investigate the mechanisms of tumorigenesis and, particularly, the mechanism by which viral proteins induce tumor progression. Viral proteins have been shown to influence not only the viral-infected cancer cells, but also the stromal cells and matricellular proteins that constitute the extracellular matrix that surrounds tumor tissues. In this review, we summarize the recent progress on the involvement of matricellular proteins in oncogenic virus-induced cancers to elucidate the mechanism of oncogenesis and consider the possible role of matricellular proteins as therapeutic targets in virus-induced cancers.

## 1. Introduction

Matricellular proteins exhibit different phenotypes to those of the classical extracellular matrix (ECM) proteins in vivo; for instance, they induce cell motility rather than providing scaffolds for stable cell adhesion [1]. In addition, they are transiently expressed as soluble proteins during embryogenesis and organogenesis and in pathological conditions rather than being constitutively expressed [1]. Interestingly, an elevated expression of these molecules has been described in various inflammatory diseases; for instance, acute viral infections are known to induce the expression of certain matricellular proteins, such as osteopontin [2]. Moreover, an altered expression of the matricellular proteins has also been detected in patients with cancers, suggesting the involvement of these molecules in tumorigenesis. It is well-known that chronic virus infections frequently cause tumors. So far, seven human tumor viruses have been identified, namely, Epstein–Barr virus (EBV), hepatitis B virus (HBV), hepatitis C virus (HCV), human papillomavirus (HPV), human T-cell leukemia virus type I (HTLV-I), and Kaposi’s sarcoma-associated herpesvirus (KSHV), and the recently characterized Merkel cell polyomavirus (MCPyV). These viruses are the etiological agents for nasopharyngeal carcinoma, HBV-related hepatocellular carcinoma (HCC), HCV-related HCC, cervical cancer, adult T-cell leukemia (ATL), Kaposi’s sarcoma (KS)/primary effusion lymphoma (PEL), and Merkel cell carcinoma (MCC), respectively [3]. Research on these virus-induced cancers has focused on the viruses or tumor antigens/tumor-derived molecules. However, it remains unclear how the host stromal cells and the matricellular proteins constituting the ECM in the tumor microenvironment are involved in the development and progression of such virus-induced cancers. Here, we focus on the involvement of the matricellular proteins in virus-induced cancers and their associations with viral oncogenes. Furthermore, we discuss the potential utilities of the matricellular proteins as therapeutic targets in virus-induced cancers. To the best of our knowledge, this is the first systematic review to describe the relationships between oncogenic human virus-induced cancers and matricellular proteins.

## 2. Current Knowledge Regarding the Roles of the Matricellular Proteins That Constitute the ECM in the Tumor Microenvironment

### 2.1. Osteopontin (OPN)

**Figure 1:**
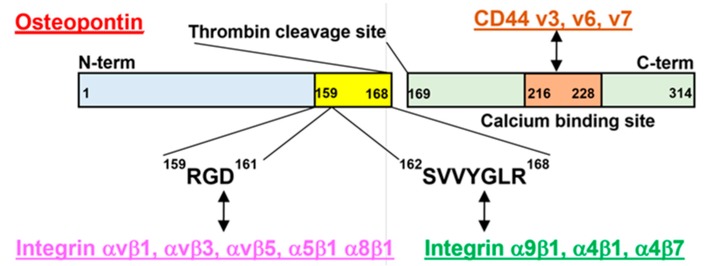
Structure of osteopontin (OPN). Amino acid (a.a.) sequences/domains involved in the receptor binding are shown. The αvβ1, αvβ3, αvβ5, α5β1, and α8β1 integrins bind to the RGD (159–161 a.a.) motif, while the α9β1, α4β1, and α4β7 integrins bind to the SVVYGLR (162–168 a.a.) motif of OPN. The CD44v3, v6, and v7 bind to the OPN via its C-terminus fragment (169–300 a.a.) including calcium binding site (216–228 a.a.).

OPN is a phosphorylated glycoprotein that is produced by several types of cells such as osteoclasts, endothelial cells, epithelial cells, and immune cells to play a major role in normal physiological processes, including bone remodeling, vascularization, and immune regulation [4]. OPN was initially named “secreted phosphoprotein 1” because it was observed to be secreted from mammalian cells that had been transformed with tumor-promoting viruses [5]. Further studies have revealed that OPN contains several variants that are encoded by alternative splicing, and the expression of each of the variants correlates with specific types of cancer [6]. OPN interacts with integrins. The integrins consist of 18 α-subunits and 8 β-subunits, and they form heterodimers consisting of an α-subunit and a β-subunit, which act as functional receptors [7]. Within OPN, the αvβ1, αvβ3, αvβ5, α5β1, and α8β1 integrins bind to the RGD motif, while the α9β1, α4β1, and α4β7 integrins bind to the SVVYGLR motif (Figure 1) [1]. OPN also binds to CD44 splice variants, such as CD44v3, CD44v6, and CD44v7, via its C-terminus fragment, including calcium-binding domain (Figure 1) [8]. Many papers have described the roles of OPN binding to integrins and CD44 in inflammatory disorders, autoimmune diseases, and tumorigenesis [4]. OPN production is elevated in various disorders; thus, it can be a useful biomarker [9]. In addition, an elevated level of anti-OPN autoantibodies has been observed in some cases [10,11]. OPN may be an attractive target for cancer therapy, and anti-OPN monoclonal antibodies (mAbs) have been evaluated in clinical models of various disorders [12,13,14,15].

### 2.2. Tenascin-C (TNC)

The tenascin family consists of 4 genes encoding TNC, tenascin-R, tenascin-X, and tenascin-W, which share biological functions in the ECM [16]. Among them, TNC is particularly interesting. TNC is a hexameric glycoprotein that consists of epidermal growth factor-like repeats, fibronectin-type 3 repeats, and a fibrinogen-related domain [17]. Through each of its domains, TNC physiologically interacts with the αvβ1, αvβ3, αvβ6, α2β1, α7β1, α8β1, and α9β1 integrins [18] and plays roles in cell motility, survival and proliferation [19]. TNC has been shown to not only regulate embryogenesis but also to be transiently expressed during inflammation or pathological responses as well as repair processes after tissue injury [20]. TNC expression is detected in stromal cells, such as fibroblasts and epithelial cells [21]. In addition, TNC is highly expressed in cancer tissues [21]. An increased concentration of TNC in plasma or serum has been detected in patients with various disorders, including cancers; thus, the expression of TNC may be a biological marker for certain diseases [22,23,24]. In addition, an elevated level of anti-TNC antibodies has been observed in some cases [25]. TNC could be an attractive target for the therapy of certain diseases, and the antigen-binding or single-chain Fv fragments derived from anti-TNC mAbs have been evaluated in heart diseases [26,27].

### 2.3. Thrombospondin (TSP)

TSP consists of the subgroup A proteins TSP-1 and -2, which form homotrimers, and the subgroup B proteins TSP-3, -4, and -5, which form homopentamers [28]. Among the TSP proteins, TSP-1 was initially isolated from human platelets as an inhibitor of angiogenesis [29]. TSP-1 consists of an N-terminal heparin-binding domain, the procollagen domain, types 1–3 repeated sequence motif regions, and the globular C-terminal domain. TSP-1 binds to the αvβ3, αIIbβ3, α3β1, α4β1, and α5β1 integrins to exert the functions of cell adhesion and migration [30]. Furthermore, it interacts with CD36 and CD47 via its type 1 central repeats and C-terminal domain, respectively; these interactions have been well studied in the regulation of angiogenesis, in which TSP-1 antagonizes the effect of the proangiogenic nitric oxide signaling pathway on endothelial cells [31]. Reduced levels of TSP-1 may be a predictor of cancer and angiogenesis-associated disease progression [32,33,34]. Thus, blocking the interaction of vascular endothelial growth factor (VEGF) with its receptor by TSP-1 mimetic during angiogenesis could be a potential approach for cancer therapy [35]. Indeed, TSP-1 peptide mimetic covalently linked to a humanized antibody have been evaluated in pre-clinical and clinical trials [36,37].

### 2.4. Periostin (POSTN)

POSTN (also known as osteoblast-specific factor 2) was originally found in osteoblasts [38]; thus, it plays a critical role in the maintenance and development of bone tissues [39]. POSTN is composed of a cysteine-rich region and a four-coiled fasciclin-like repeats region [40]. In addition to interacting with the matricellular proteins described above, POSTN also interacts with the αvβ3, αvβ5 and α6β4 integrins to promote the invasion of tumor cells [41,42]. In addition, POSTN is known to activate the canonical wingless-related integration site (Wnt) signaling pathways, which are involved in cancer stem cell maintenance [43]. Related to this role in the regulation of Wnt signaling, POSTN has been identified as an epithelial-mesenchymal transition (EMT) marker [44]. Thus, these two independent interaction-induced signaling pathways are important for the development and maintenance of cancer cells [40]. Elevated levels of POSTN have been detected in sera from patients with various types of cancers [45,46,47], suggesting that POSTN may be a useful biomarker for those cancers. The detection of POSTN by the immunohistochemical (IHC) staining of tumor lesions by anti-POSTN mAbs targeting the fasciclin domain, which can inhibit integrin-mediated cell migration in vitro, has also been reported [48]. In a breast tumor model in vivo, the administration of anti-POSTN neutralizing antibodies inhibited the proliferation, migration, and invasion of breast tumor cells, as well as the differentiation of osteoclasts [49].

### 2.5. Secreted Protein Acidic and Rich in Cysteine (SPARC)

SPARC (also known as osteonectin and basement membrane 40) is a secreted glycoprotein that was initially identified as a bone-specific protein showing selective binding to collagen and hydroxyapatite [50]. SPARC consists of three functional domains including an N-terminus acidic domain, a follistatin-like domain, and a C-terminus domain [51]. Cell type secreting SPARC include endothelial cells, fibroblasts, pericytes, astrocytes, osteoblasts, and macrophages in regulating several physiological processes, including development, tissue remodeling, and wound repair [52]. SPARC is also involved in the development of fibrosis in various disorders including hypertension, diabetes, and liver cirrhosis [52]. In an in vivo model using SPARC-deficient mice, SPARC exhibited a tumor suppressor function modulating carcinogenesis, progression, and metastasis [53]. Interestingly, however, the overexpression of SPARC was observed in certain types of tumors [54]; thus, it may act as an indicator of tumor progression [55]. The IHC staining of prostate cancer samples showed the presence of SPARC protein in the epithelial tumoral cells of metastatic cases, indicating that it may promote the invasion and metastasis of cancer cells [56]. In lung cancer, transforming growth factor (TGF)-β1-induced extracellular signal-regulated kinase (ERK) signaling enhanced the expression of SPARC mRNA [57]. Correspondingly, SPARC expression in tumor cells correlated with EMT features, and the expansion and infiltration of myeloid-derived suppressor cells, which contribute to tumor growth and dissemination, was indirectly regulated by SPARC, as well as OPN [58].

## 3. The Involvement of Matricellular Proteins in the Tumorigenesis of Oncogenic Virus-Induced Cancers and Their Potential Utilities as Therapeutic Targets

So far, seven viruses have been well characterized as the causative agents of specific human cancers [3]. Although numerous papers have reported on the mechanisms of the tumorigenesis of oncogenic virus-induced cancers, it remains unclear whether and how the matricellular proteins are involved in the tumorigenesis of those cancers. In this section, we summarize the reports describing the expression and production of matricellular proteins in the oncogenic virus-induced cancers. In Section 3.2, Section 3.3, Section 3.4, Section 3.5, Section 3.6 and Section 3.7 we discuss the reports describing only virus-infection-related cancer cases, excluding non-viral-related cases and those in which the presence of a viral infection was unclear.

### 3.1. HTLV-I-Induced ATL

**Figure 2:**
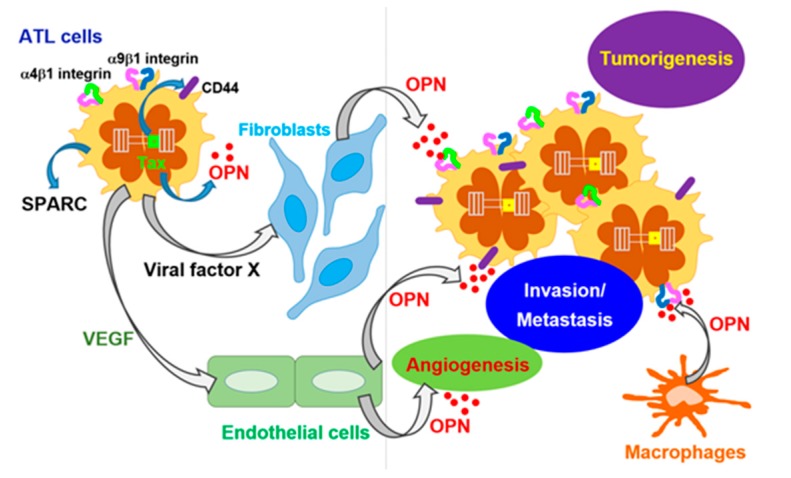
Proposed mechanisms of adult T-cell leukemia (ATL) tumorigenesis and metastasis.

HTLV-I is classified in the *Retroviridae* family; the HTLV-I genome comprises approximately 9 kb of single-stranded RNA encoding three structural genes (*gag*, *pol*, and *env*) and several alternatively-spliced regulatory genes [59]. HTLV-I has been identified as a causative agent of ATL, which is a CD4^+^ T-cell malignancy [60]. Recent studies have revealed the genetic and epigenetic changes that are involved in ATL tumorigenesis [61,62]. The viral factors involved in ATL oncogenesis are Tax and HBZ [59]. The expression of OPN was partially induced by the HTLV-I Tax protein [63]. However, the expression of OPN in ATL cell lines and HTLV-I-infected T cell lines is low [64]. The IHC staining of tissues from ATL patients revealed the expression of OPN in both tumors and stromal cells such as macrophages and endothelial cells [64,65]. Furthermore, in the plasma of ATL patients, the OPN levels detected by enzyme-linked immunosorbent assay (ELISA) were elevated and showed a correlation with the disease severity, suggesting that OPN is critically involved in the development of ATL [65]. In the NOD/Shi-*scid*,*IL-2Rg^null^* (NOG) mouse model transplanted with ATL cells, the expression of host stromal cell-derived OPN increased during the progression of the disease, indicating that the stromal OPN may support the tumorigenesis of ATL (Figure 2) [64].

The mechanism of elevated OPN expression from the host cells is not yet clear. It is intriguing that OPN production can be detected in the fibroblasts of ATL patients [64] and the co-cultivation of ATL cells with mouse embryo-derived fibroblasts enhanced their OPN production in vitro. Thus, the molecular mechanisms of OPN induction by viral factor X(s) are currently being investigated (Figure 2). Considering the potential therapeutic aspect, anti-OPN mAbs exhibited suppressive effects on ATL tumor growth and metastasis by suppressing OPN expression in cancer-associated fibroblasts [64]. In addition, OPN is known to induce VEGF via the activation of phosphoinositide 3-kinase (PI3K)/protein kinase B (Akt) and ERK1/2 signaling in endothelial cells [66], and it promotes VEGF-dependent breast tumor growth and angiogenesis [67]. Thus, the elevated expression of OPN in ATL may be involved in angiogenesis. At least two reports have indicated that VEGF is a critical factor for the progression of ATL [68,69]. However, the expression of VEGF is Tax-independent [70], suggesting that the role of OPN-induced VEGF expression in the tumorigenesis of ATL warrants further investigation. As mentioned in Section 2.5, SPARC exhibited a tumor suppressor effect [51]. Interestingly, the use of RNA interference to suppress the expression of SPARC enhanced the apoptotic effects of bortezomib on ATL cells by increasing cleaved caspase 3 expression, suggesting that SPARC could be another therapeutic target for ATL (Table 1) [71].

### 3.2. HBV-Related HCC

HBV is a member of the *Hepadnaviridae* family. The HBV genome comprises approximately 3.2 kb of double-stranded DNA encoding seven proteins and four regulatory elements [72]. While the mechanism of HCC tumorigenesis by HBV has been thought to be caused by an immune response leading to chronic inflammation, the HBV x protein (HBx) has also been considered to be involved in HCC tumorigenesis [73]. The HBx can upregulate the expression of OPN via 5-lipoxygenase (5-LOX) in vitro (Table 1) [74,75]. The concentration of OPN in the blood plasma is predictive of cirrhosis in patients with HBV infection [76,77]. IHC staining also confirmed the relationship between OPN and HBV-related HCC [78], suggesting that OPN is involved in several steps during the development of HCC in the HBV-infected liver. The administration of OPN during the immune response against HBV improved the maturation and function of dendritic cells, suggesting the possibility of using immunotherapy for HCC [79]. A recent study identified that TSP-1 was upregulated in the sera of patients with hepatic fibrosis by an unknown mechanism (Table 1) [80].

### 3.3. HCV-Related HCC

HCV is classified in the *Flaviviridae* family. The HCV genome comprises approximately 9.6 kb of single-stranded positive sense RNA encoding three structural proteins and seven non-structural proteins [81]. The HCV genes encoding proteins with oncogenic potential are not identified. Thus, similar to HCC tumorigenesis induced by HBV, that induced by HCV has been also considered to result from an immune response followed by chronic inflammation [82]. In HCV-infected cells, two nonstructural proteins encoded by HCV, namely NS3/4A and NS5A, played critical roles in the induction and secretion of TGF-β1 (Table 1) [83]. HCV induces the intracellular expression of TSP-1, which is involved in the proteolytic activation of TGF-β1 [83]. The HCV core protein was also reported to mediate the activation of TGF-β via TSP-1 (Table 1) [84]. TGF-β signaling can induce EMT- and tumor-initiating cancer stem-like cells in hepatocytes [85,86,87]; thus, such HCV core protein-induced TSP-1-mediated TGF-β1 activation is a critical event in HCC tumorigenesis. Serum or plasma OPN could be a biomarker for hepatic fibrosis or inflammation in patients infected with HCV [88,89,90,91]. Therefore, OPN is a biomarker for HCC development. Antisense oligodeoxynucleotides (ODNs) suppressing the expression of OPN inhibited the migration and invasion of an HCC cell line in vitro. Furthermore, when nude mice were transplanted with the same HCC cell line in vivo, the antisense ODNs targeting the tumor-derived expression of OPN significantly suppressed lung metastasis via downregulating matrix metalloproteinase (MMP)-2 and urokinase-type plasminogen activator (uPA) (Table 1) [92]. These findings suggest that OPN is a therapeutic target for suppressing the metastasis of HCC. The molecular mechanism of HCV-induced OPN processing involves the increased phosphorylation and activation of p38, c-Jun N-terminal kinase, phosphatidylinositol 3-kinase, and mitogen-activated protein kinase kinase 1/2 [93].

### 3.4. HPV-Induced Cervical Cancer

HPV is classified in the *Papillomaviridae* family. The HPV genome comprises approximately 8 kb of double-stranded DNA, which encodes six early genes and two late genes [94]. The HPV genes encoding the E6 and E7 proteins have been considered as oncogenes involved in the tumorigenesis of cervical cancer [95]. The experimental expression of the E6 and E7 proteins of HPV-16 increased the expression of VEGF/interleukin-8 and decreased that of TSP-1 in keratinocytes (Table 1) [96,97,98], suggesting that those HPV proteins are involved in angiogenesis. Bao et al. reported that OPN was highly detected in HPV-positive patients with cervical cancer, suggesting that OPN could be involved in the HPV-associated progression of cervical cancer [99]. However, that study did not describe any relationship between the HPV proteins and the enhanced OPN expression in HPV-positive lesions. The HPV minor capsid protein L2 is cleaved by furin, which is a cellularly-encoded proprotein convertase that is required for endosome escape [100]. OPN was reported to induce the expression of furin via p38 and nuclear factor κB signaling and thereby support the progression of cervical cancer [101], suggesting that the OPN-furin axis is involved in not only the viral entry step but also the pathogenesis of cervical cancers (Table 1). Enhanced Tenascin expression was detected in cervical and vulvar koilocytotic lesions [102]. In addition, Tenascin mRNA was detected in cervical lesions and its expression level strongly correlated with the degree of inflammation but not that of dysplasia, indicating that tenascin could be a marker of the premalignant stage of cervical cancer (Table 1) [103]. The infected cells undergo a process of differentiation during the viral life cycle. Thus, achieving a fuller understanding the interactions between the infected cells and the stromal cells in the tumor microenvironment should be regarded as a critical goal [104].

### 3.5. EBV-Related Nasopharyngeal Carcinoma

EBV (also known as human herpesvirus 4) is classified in the *Herpesviridae* family. The EBV genome comprises approximately 170 kb of double-stranded DNA [105]. It is the first human tumor virus that was reported to be involved in Burkitt lymphoma, which is an aggressive B-cell malignancy, and it has been also identified as a causative agent for nasopharyngeal carcinoma [106]. A gene expression profiling analysis confirmed the elevated expression of OPN and SPARC in EBV-infected gastric adenocarcinoma (Table 1) [107]. The expression of SPARC and POSTN was found to be upregulated in nasopharyngeal carcinoma stem cells and showed a correlation with the expression of EBV genes such as EBV-encoded small RNA (EBER) and latent membrane protein 1 (LMP1) (Table 1) [108]. However, a role of these matricellular proteins in the pathogenesis of nasopharyngeal carcinoma remains to be elucidated.

### 3.6. KSHV-Related KS and PEL

KSHV (also known as human herpesvirus 8) is also classified in the *Herpesviridae* family. The KSHV genome comprises approximately 140 kb of double-stranded DNA [109]. KSHV infection is thought to be a causative factor of KS and PEL, in which the cells that give rise to the tumors are epithelial cells and B cells, respectively [110,111]. The IHC staining of KS lesions revealed that tenascin (of an unspecified subtype) was expressed in the vessel walls within, and outside, the spindle cell compartment, while TSP was expressed only by endothelial cells inside and outside the lesion, but not by the spindle cells (Table 1) [112]. TSP-1 has shown to inhibit VEGF signaling [31] and to suppress the induction of endothelial cell proliferation and motility by the conditioned supernatant of KS-like cells [113]. In a microarray analysis, the reduction of TSP-1 expression by a viral microRNA (miRNA) decreased the activity of TGF-β, which is a potent anti-inflammatory factor (Table 1) [114]. In PEL, the ceramide-induced upregulation of TSP-1 influenced the expression of multiple KSHV-related miRNAs and thereby led to cell cycle arrest [115]. Taken together, these observations collectively support the concept that TSP-1 regulates the angiogenic responses in KS lesions and PEL. In the microarray analysis, the expression of OPN was also decreased by the viral miRNA, although that study did not examine the involvement of OPN in angiogenesis and tumorigenesis [114].

### 3.7. MCPyV-Induced MCC

MCPyV has been recently identified as a causative agent of MCC, a neuroendocrine carcinoma of the skin [116]. The MCPyV genome comprises approximately 5.4 kb of double-stranded DNA, which is divided into three major regions: the non-coding regulatory region, the early coding region, and the late coding region [117]. A study performed IHC staining for TNC in MCC lesions and reported that TNC was expressed at metastatic areas and its abundance correlated with tumor size (Table 1) [118,119]. Although that study did not confirm the occurrence of MCPyV infection in the samples (since MCPyV had not yet been identified as a causative agent of MCC when that study was performed), their observations on the IHC staining of TNC are intriguing. Liu et al. reported that dermal fibroblasts are the natural host cells that support productive MCPyV infections ex vivo [120]. In the dermal fibroblasts, MMP gene expression and Wnt/β-catenin signaling were induced by MCPyV infection. The MMPs are thought to play important roles in tumor invasion and metastasis; thus, an elevated expression of MMPs may contribute to the progression of MCC. MCPyV encodes the small T (sT) antigen, which is an oncogene that transforms rodent fibroblasts [121]. However, it is currently unclear whether any relationships between the sT antigen and matricellular proteins contribute to the tumorigenesis of MCC.

## 4. Conclusions

The regulatory functions of matricellular proteins in the oncogenesis of virus-induced cancers and their potential utilities as therapeutic targets for such cancers has been summarized above. An altered abundance of certain matricellular proteins has frequently been detected in samples from patients with virus-induced cancers. The matricellular proteins are well evidenced to be involved in the tumorigenesis of such cancers, depending not only on the cell type, but also on the expression of viral oncogenes and their associated molecules in the cancer cells. However, based on this review of the recent reports, it seems that the potential involvement of the matricellular proteins in viral tumorigenesis has not been fully investigated. In particular, there is a lack of information regarding the involvement of the surrounding stromal cells and secreted matricellular proteins in the tumor microenvironment. Although the analysis of plasma or serum samples by ELISA is useful for determining the circulating concentrations of the matricellular proteins, this method cannot identify which cell type is the source of the proteins. Thus, the IHC staining of tumor lesions is more informative. Furthermore, a lack of suitable animal models for human oncogenic virus infections and the tumor development associated with those infections has hampered the progress of research on virus-induced cancers. Therefore, animal models that closely mimic the human virus-induced cancers need to be established. Based on our research on OPN and integrins in the ATL xenograft NOG mouse model [64], we believe that the involvement of matricellular proteins in human virus-induced cancers can be investigated by distinguishing between the tumor-derived human proteins and host stromal cell-derived murine proteins in vivo. Through our research, we have found that the interactions of tumor cells with host stromal cell-derived matricellular proteins are attractive as potential therapeutic targets. Thus, the development of mAbs and/or their genetically-engineered derivatives, such as their antigen-binding and single-chain Fv fragments targeting such interactions is encouraged [122]. Further investigations will be necessary to elucidate the clinical relevance and application of matricellular proteins in human oncogenic virus-induced cancers.

## Figures and Tables

**Table 1:** Association of viral genes/oncogenes with the matricellular proteins.

Viruses	Viral Genes/Oncogenes	Matricellular Proteins	Phenomena/Mechanisms	References
HTLV-I	Tax	OPN	Transcriptional upregulation of OPN resulted in the activation of PI3K/Akt pathway	[63]
	?	OPN	Stromal cell-derived OPN involved in the tumorigenesis and metastasis	[64,65]
	?	SPARC	SPARC inhibition resulted in caspase 3-dependent apoptosis by bortezomib	[71]
HBV	HBx	OPN	5-LOX-dependent upregulation of OPN promoted cell migration	[74,75]
	?	OPN	Elevated production in cirrhosis and HCC	[76,77,78]
	?	TSP-1	Elevated production in fibrosis	[80]
HCV	NS3/4A, NS5A	TSP-1	Proteolytic activation of TGF-β by intracellular TSP-1	[83]
	Core	TSP-1	Increased secretion of TSP-1 activated TGF-β	[84]
	?	OPN	Antisense ODNs suppressed lung metastasis via downregulating MMP-2 and uPA	[92]
HPV	E6, E7	TSP-1	Decreased expression in keratinocytes	[96,97,98]
	?	OPN	Induction of furin via p38 and NF-kB resulted in cancer progression	[99]
	?	Tenascin *	Expressed during the premalignant stage	[103]
EBV	?	OPN	Elevated expression	[107]
	LMP1, EBER	SPARC POSTN	Elevated expression	[107,108]
KSHV	?	Tenascin *	Expressed in the vessel walls	[112]
	?	TSP-1	Viral miRNA-dependent TSP-1 reduction decreased TGF-β activity	[113,114]
	?	OPN	Viral miRNA-dependent reduction	[114]
MCPyV	sT antigen?	TNC	Tumorigenesis and metastasis	[118,119]

* An unspecified subtype; Question marks indicate that the relationships between viral genes/oncogenes and matricellular proteins are unclear.

## Abbreviations

a.a.	Amino acid
Akt	Protein kinase B
ATL	Adult T-cell leukemia
EBER	Epstein–Barr virus-encoded small RNA
EBV	Epstein–Barr virus
ECM	Extracellular matrix
ELISA	Enzyme-linked immunosorbent assay
EMT	Epithelial-mesenchymal transition
ERK	Extracellular signal-regulated kinase
HBV	Hepatitis B virus
HBx	Hepatitis B virus x protein
HCC	Hepatocellular carcinoma
HCV	Hepatitis C virus
HHV	Human herpesvirus
HPV	Human papillomavirus
HTLV-I	Human T-cell leukemia virus type I
IHC	Immunohistochemical
KS	Kaposi’s sarcoma
KSHV	Kaposi’s sarcoma-associated herpesvirus
LMP1	Latent membrane protein 1
LOX	Lipoxygenase
mAb	Monoclonal antibody
MCPyV	Merkel cell polyomavirus
MCC	Merkel cell carcinoma
MEK	Mitogen-activated protein kinase kinase
miRNA	MicroRNA
MMP	Matrix metalloproteinase
NOG	NOD/Shi- *scid*,*IL-2Rg^null^*
ODN	Oligodeoxynucleotide
OPN	Osteopontin
PEL	Primary effusion lymphoma
PI3K	Phosphoinositide 3-kinase
POSTN	Periostin
SPARC	Secreted protein acidic and rich in cysteine
sT	Small T
TGF-β	Transforming growth factor-beta
TNC	Tenascin-C
TSP	Thrombospondin
uPA	Urokinase-type plasminogen activator
VEGF	Vascular endothelial growth factor
